# Evidence for the evolving role of neoadjuvant and perioperative immunotherapy in resectable non-small cell lung cancer

**DOI:** 10.37349/etat.2024.00273

**Published:** 2024-09-29

**Authors:** Thomas Hansen, Jonathon Hill, Gary Tincknell, Derrick Siu, Daniel Brungs, Philip Clingan, Lorraine Chantrill, Udit Nindra

**Affiliations:** King’s College London, UK; IRCCS Istituto Romagnolo per lo Studio dei Tumori (IRST) “Dino Amadori”, Italy; ^1^Department of Medical Oncology, St George Hospital, Kogarah, Sydney 2217, NSW, Australia; ^2^Department of Medical Oncology, Wollongong Hospital, Illawarra Shoalhaven Local Health District, Illawarra 2500, NSW, Australia; ^3^Cancer Care Wollongong, Wollongong 2500, NSW, Australia; ^4^Graduate School of Medicine, University of Wollongong, Wollongong 2500, NSW, Australia; ^5^School of Chemistry and Molecular Biosciences, University of Wollongong, Wollongong 2500, NSW, Australia; ^6^National Health and Medical Research Council Clinical Trials Centre, University of Sydney, Camperdown 2050, NSW, Australia; ^7^Department of Medical Oncology, Liverpool Hospital, South Western Sydney Local Health District, Liverpool 2170, NSW, Australia

**Keywords:** Chemoimmunotherapy, resectable NSCLC, pathological complete response, overall survival, surgery

## Abstract

The treatment of early-stage non-small cell lung cancer (NSCLC) is becoming increasingly complex. Standard of care management for the past decade has been adjuvant chemotherapy following curative intent resection regardless of nodal status or tumour profile. With the increased incorporation of immunotherapy in NSCLC, especially in the locally advanced, unresectable, or metastatic settings, multiple studies have sought to assess its utility in early-stage disease. While there are suboptimal responses to neoadjuvant chemotherapy alone, there is a strong rationale for the use of neoadjuvant immunotherapy in tumour downstaging, based upon the concept of enhanced T cell priming at the time of a high tumour antigen burden, and demonstrated clinically in other solid tumours, such as melanoma. In the NSCLC cancer setting, currently over 20 combinations of chemoimmunotherapy in the neoadjuvant and perioperative setting have been studied with results variable. Multiple large phase III studies have demonstrated that neoadjuvant chemoimmunotherapy combinations result in significant advances in pathological response, disease free and overall survival which has led to practice change across the world. Currently, combination immunotherapy regimens with novel agents targeting alternate immunomodulatory pathways are now being investigated. Given this, the landscape of treatment in resectable early-stage NSCLC has become increasingly complex. This review outlines the literature of neoadjuvant and perioperative immunotherapy and discusses its potential benefits and complexities and ongoing considerations into future research.

## Introduction

Lung cancer is the leading cause of cancer-related mortality, accounting for approximately 1.8 million deaths annually [1]. Non-small cell lung cancer (NSCLC) is the commonest subtype, representing approximately 80% of lung cancers. In early-stage NSCLC, curative surgical resection remains the cornerstone of management. However, NSCLC is surgically resectable in about 25% of patients at time of diagnosis [2]. Currently, criteria for resectability in early-stage NSCLC vary by institution but generally include stage I to IIIA disease. Patients may have ipsilateral hilar nodal involvement but evidence of contralateral lung or nodal disease exclude patients from curative therapy. Furthermore, long-term outcomes following surgical resection, particularly for those with pathologically proven advanced nodal disease are poor, and the use of adjuvant chemotherapy only confers an approximate 5% improvement in 5-year overall survival (OS) [3].

**Figure 1:**
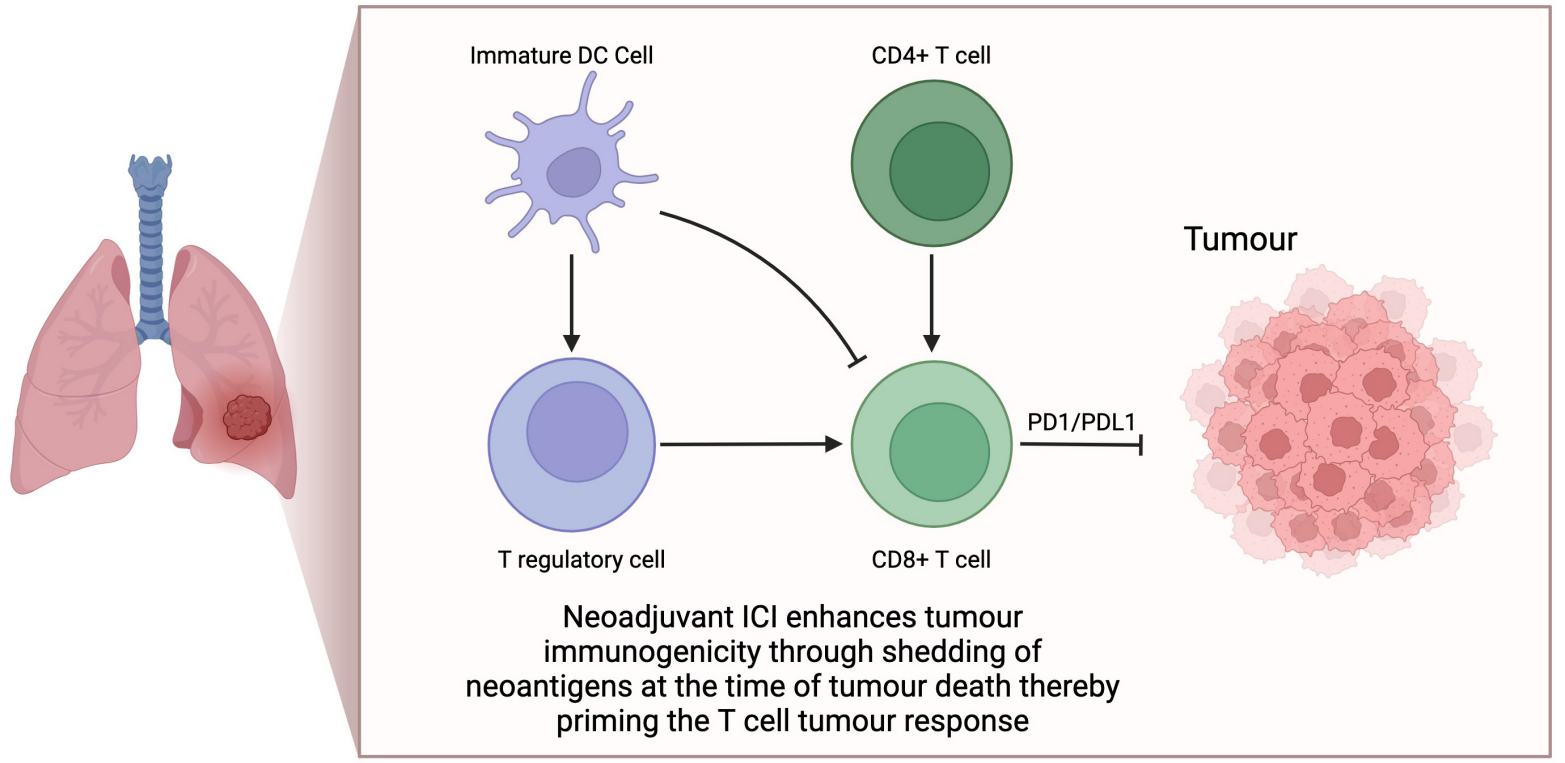
Neoadjuvant immune-checkpoint inhibitor activation in non-small cell lung cancer. Neoadjuvant immune checkpoint inhibitors (ICIs) can enhance T-cell priming through T-regulatory and immature dendritic cell pathways. Removal of the tumour during surgery is suspected to augment the response of tumour infiltrating lymphocytes, thereby potentially reducing the efficacy of ICIs. This forms the rationale for ongoing studies in the neoadjuvant lung cancer treatment pathway. Created in BioRender. Nindra, U. (2024) BioRender.com/w52g417

In the locally advanced, unresectable, and metastatic settings, immunotherapy now plays a central role in standard of care management. This is driven by the improved outcomes seen in the PACIFIC study [4] with consolidation durvalumab in stage III unresectable patients, and the KEYNOTE-189 [5], CheckMate-227 [6] and IMPOWER-150 [7] studies in the metastatic setting. As a result, there has been a significant paradigm shift for the earlier incorporation of immunotherapy in NSCLC treatment. The theoretical benefits of a neoadjuvant IO approach in NSCLC are well documented. It is generally accepted that the presence of a full tumour mass upon commencement of immunotherapy enhances tumour immunogenicity through shedding of neoantigens at time of tumour cell death, thereby “priming” the T-cell tumour response as outlined in the schematic in Figure 1 [8]. Upfront resection of the tumour and associated tumour infiltrating lymphocytes (TILs) is therefore likely to negatively impact the intensity of the T-cell response that would be otherwise sustained from PD1/PD-L1 agents. Certainly, the augmented response of TIL activation using neoadjuvant treatment, compared to adjuvant therapy, is clearly demonstrated in pre-clinical breast cancer models [9]. Recently, this has been replicated in the clinical setting in resectable stage IIIB–IV melanoma, where neoadjuvant immunotherapy followed by surgery and adjuvant immunotherapy demonstrated greater clinical disease-free survival compared with surgery plus adjuvant immunotherapy alone [10, 11]. This was despite an identical duration of treatment in both arms. Currently pre-clinical models in lung and other solid cancer also support similar conclusions of improved TIL invasion into sites of disease and greater CD8 positive T-cell responses.

Given these strong rationales for the use of neoadjuvant immunotherapy there has been burgeoning interest in exploring its utility in the neoadjuvant space. CheckMate-816 was a landmark trial in this context, demonstrating that the addition of neoadjuvant nivolumab to chemotherapy resulted in significantly longer event-free survival and increased pathological complete response (pCR) compared to chemotherapy alone [12]. This study led to the first regulatory approval through the FDA of neoadjuvant chemoimmunotherapy in early non-small lung cancer, and paved the way for a multitude of further trials since investigating novel neoadjuvant immunotherapeutic approaches.

In light of this, the available evidence of immune-therapeutics in the neoadjuvant lung landscape is rapidly changing. We herein present a review of the currently reported evidence using neoadjuvant and perioperative approaches in resectable NSCLC. In addition, an insight into available predictive and prognostic biomarkers and the potential future directions of neoadjuvant immunotherapy in the precision medicine era. The following sections will highlight the current clinical trials that have been completed or are ongoing in the lung cancer space and their key results.

Our preliminary search identified 1,464 articles from which duplicates were removed and 1,166 were screened. 136 articles were retrieved for full review. Overall, 25 articles were included in the final analysis. We stratified the articles into four subgroups. This included neoadjuvant immunotherapy with chemotherapy, neoadjuvant immunotherapy plus radiotherapy, neoadjuvant immunotherapy alone, and perioperative chemo-immunotherapy. Data regarding treatment protocols, major pathological response (MPR), pCR, and survival data where available were extracted and are summarised in Table 1.

**Table 1:** Summary of studies investigating neoadjuvant immunotherapy in resectable lung cancer

**Study**	**Author, year**	**Study phase**	**Study protocol**	**Study population**	**Number of patients**	**MPR (%)**	**pCR (%)**	**DFS/EFS/OS (%)**
Trials investigating neoadjuvant immunotherapy + chemotherapy								
NeoSTAR NCT03158129	Cascone et al. [14], 2023	II	Arm 1: Nivolumab (x3) Arm 2: Nivolumab + Ipilimumab (x1) Arm 3: Nivolumab (x3) + CT Arm 4: Nivolumab (x3) + Ipilimumab (x1) + CT	Resectable IA-IIIA NSCLC	Arm 1: 23 Arm 2: 21 Arm 3: 22 Arm 4: 22	22 38 32.1 50	9 29 18.2 18.2	12 m EFS: 96% (Arm 3) vs. 82% (Arm 4)
CheckMate-816 NCT02998528	Spicer et al. [13], 2023	III	Arm 1: Nivolumab + Platinum CT Arm 2: Platinum CT	Resectable IB-IIIA NSCLC	Arm 1: 179 Arm 2: 179	36.9 8.9	24 2.2	31.6 m (95% CI, 30.2-NR) vs. 20.8 m (95% CI, 14.0 to 26.7) HR 0.63; *P* = 0.005). 24 m EFS: 63.8% vs. 45.3% OS improvement of 13% in Nivolumab + CT group
No NCT IDN	Shen et al. [15], 2021	I	Pembrolizumab 2 mg/kg + Nab-paclitaxel + Carboplatin (x2)	Resectable stage IIB–IIIB squamous lung	37	45.9	64.9	-
NCT04304248	Zhao et al. [40], 2021	II	Toripalimab + Carboplatin + Pemetrexed/Nab-paclitaxel (x3)	Resectable stage IIIA or T3-4N2 IIIB NSCLC	33	60.6	45.5	-
Lung-Mate001 ChiCTR1900023758	Zhang et al. [37], 2021	II	Sintilimab + Carboplatin + Pemetrexed/Gemcitabine) (x2-4)	Resectable stage IB–IIIA	50	43.3	20	-
Neo-Pre-IC NCT04326153	Sun et al. [41], 2024	II	Sintilimab + Nab-paclitaxel + Carboplatin (x2-3)	Resectable IIIA/IIIB NSCLC	30	65	40	24 m DFS: 75%
No NCT	Duan et al. [42], 2021	I	CT + Immunotherapy	Stage IIA–IIIB NSCLC	23	50	30	-
NCT02716038	Shu et al. [43], 2020	II	Atezolizumab + Carboplatin/Nab-paclitaxel (x2-4)	Resectable IB-IIIA NSCLC	30	57	33	-
NCT03480230	Tfayli et al. [16], 2020	I	Avelumab (x4) + Cisplatin/Carboplatin + Gemcitabine/Pemetrexed (x3)	Stage IB–IIIA NSCLC	15	22	11	-
Trials investigating neoadjuvant immunotherapy								
ChiCTR-OIC-17013726	Gao et al. [44], 2020	IB	Sintilimab 200 mg (x2)	Resectable stage IA–IIIB NSCLC	40	40.5	16.2	-
CheckMate-159 NCT02259621	Forde et al. [17], 2018; Reuss et al. [18], 2020	IB/II	Arm 1: Nivolumab (x2) Arm 2: Nivolumab (x3) + Ipilimumab (x1)	Resectable IB-IIIA NSCLC	Arm 1: 21 Arm 2: 9	45 33.3	10 33.3	-
NEOMUN NCT03197467	Eichhorn et al. [45], 2021	II	Pembrolizumab (x2)	Resectable stage II–IIIA	15	27	-	-
IONESCO NCT03030131	Wislez et al. [20], 2022	II	Durvalumab (x3)	IB-IIIA (non-N2) resectable NSCLC	46	19	3	12 m OS—89% (95% CI 75.8% to 95.3%) 12 m DFS—78% (95% CI 63.4% to 87.7%)
NeoCOAST NCT03794544	Cascone et al. [19], 2023	II	Arm 1: Durvalumab Arm 2: Durvalumab + Oleclumab Arm 3: Durvalumab + Monalizumab Arm 4: Durvalumab + Danvatirsen	Stage IA–IIIA NSCLC	Arm 1: 27 Arm 2: 21 Arm 3: 20 Arm 4: 16	12.5 22.2 33.3 33.3	3.7 9.5 10 12.5	-
NCT02927301	Rusch et al. [46], 2023	II	Atezolizumab (x2)	Resectable stage IB–IIIB (non-EGFR/ALK)	143	20	6	-
Trials investigating perioperative immunotherapy +/- chemotherapy								
NADIM NCT03081689	Provencio et al. [47], 2020	II	Paclitaxel + Carboplatin + Nivolumab (x3) > Sx > Nivolumab	Resectable stage IIIA NSCLC	46	83	63	24 m PFS 77.1%
NADIM II NCT03838159	Provencio et al. [25], 2022	II	Arm 1: Nivolumab + Platinum CT (x3) > Sx > Nivolumab (x6) Arm 2: Platinum CT	Resectable stage IIIA–IIIB NSCLC	Arm 1: 57 Arm 2: 29	53 14	37 7	24 m OS 85% vs. 63.6% (HR 0.43) 24 m PFS 67.2% vs. 40.9% (HR 0.47)
TOP 1501 NCT02818920	Tong et al. [48], 2022	II	Pembrolizumab (x2) > Sx > Pembrolizumab (x4)	Stage IB–IIIA NSCLC	30	28	-	-
KEYNOTE-671 NCT034256643	Wakelee et al. [24], 2023	III	Arm 1: Pembrolizumab + Platinum CT (x4) > Sx > Pembrolizumab (x13) Arm 2: Placebo + Platinum CT (x4) > Sx > Placebo (x13)	Resectable stage II–IIIB NSCLC	797	30.2 11	18.1 4	24 m EFS—62.4% vs. 40.6% (HR 0.58) 24 m OS: 80.9% vs. 77.6% (*P* = 0.02)
NEOTORCH4 NCT04158440	Lu et al. [49], 2024	III	Arm 1: Toripalimab + Platinum CT (x3) > Sx > Toripalimab (x13) Arm 2: Placebo + Platinum CT (x3) > Sx > Placebo (x13)	Resectable stage II–III NSCLC	Arm 1: 202 Arm 2: 202	48.5 8.4	24.8 1	mEFS: NE (95% CI, 24.4m–NE) vs. 15.1 m (95% CI, 10.6 m–21.9 m) (HR 0.40)
SAKK 16/14 NCT02572843	Rothschild et al. [22], 2021	II	Docetaxel + Cisplatin (x3) + Durvalumab (x2); > Sx > Durvalumab (x26)	T1-3N2 stage IIIA NSCLC	67	62	18	12 m EFS—73% 12 m OS—91%
AEGEAN NCT03800134	Heymach et al. [23], 2023	III	CT + Durvalumab or placebo (x3) > Sx > Durvalumab or placebo (x12)	Resectable Stage II–IIIB	Arm 1: 366 Arm 2: 374	33.3 12.3	17.2 4.3	EFS NR (31.9 m–NR) vs. 25.9 m (18.9 m–NR), HR 0.68
NCT04316364	Yan et al. [50], 2023	IB	Adebrelimab, Nab-paclitaxel + Carboplatin (x3) > Sx > Adebrelimab (x16)	Resectable stage II–III NSCLC	37	51.4	29.7	-
Trials investigating neoadjuvant IO with radiotherapy								
NCT02904954	Altorki et al. [51], 2021	II	Durvalumab alone vs. Durvalumab + Stereotactic RT	Resectable stage I–IIIA	Arm 1: 30 Arm 2: 30	6.7 53.3	26	-
NCT03030131 (IONESCO)	Wislez et al. [20], 2022	II	Durvalumab alone vs. Durvalumab + Stereotactic RT	Stage I–IIIA	Arm 1: 26 Arm 2: 26	-	-	-

m: month; NSCLC: non-small cell lung cancer; CT: chemotherapy; EFS: event free survival; NR: not reached; CI: confidence interval; NCT: National Clinical Trial; IDN: identification number; DFS: disease free survival; PFS: progression free survival; HR: hazard ratio; Sx: surgery; MPR: major pathological response; pCR: pathological complete response; OS: overall survival; EGFR: epidermal growth factor receptor; ALK: anaplastic lymphoma kina

## Neoadjuvant immunotherapy and chemotherapy

In total, there were nine published trials investigating the effect of combination chemotherapy with immunotherapy involving six different immunotherapy agents. In respect to trials investigating nivolumab, CheckMate-816 is the largest phase III trial to date and evaluated the effectiveness of three cycles of neoadjuvant nivolumab with platinum-based chemotherapy compared to platinum-based chemotherapy alone in 358 randomised patients [12]. Chemoimmunotherapy was associated with a significantly longer event free survival [EFS; hazard ratio (HR) 0.63] in addition to a greater proportion of patients achieving pCR (24.0% vs. 2.2%). An exploratory analysis also revealed improved EFS in the subset of patients with pCR. Updated 3-year data has promoted the durability of this treatment response, with 3-year EFS of 57% compared to 43% (HR 0.68) in the nivolumab plus chemotherapy arm vs. chemotherapy alone groups respectively [13].

The phase II NeoSTAR trial expansion arms investigated the efficacy of neoadjuvant nivolumab and doublet nivolumab with ipilimumab with chemotherapy in patients with stage I to IIIA NSCLC [14]. In the 44 patients enrolled, the rate of MPR was 32.1% in the nivolumab plus chemotherapy group and 50% in the triplet cohort. When patients with known epidermal growth factor receptor (EGFR)/anaplastic lymphoma kina (ALK) alterations were excluded, the MPR rates improved further to 41.2% and 62.5%, respectively. The addition of ipilimumab appeared to reduce the extent of residual viable tumour, an effect which was more pronounced in the subset of patients with stage IIIA disease. This suggests that a subset of patients with more advanced nodal disease may benefit from dual checkpoint inhibition and merits further investigation in larger phase III studies. Importantly, the incorporation of CTLA-4 blockade was well tolerated without significant toxicities compared to anti-PD1 monotherapy.

Chemoimmunotherapy with pembrolizumab has also been assessed in the neoadjuvant NSCLC space. In a phase I study, Shen et al. [15] assessed the efficacy of neoadjuvant pembrolizumab with doublet chemotherapy in patients with stage IIB–IIIB squamous cell lung cancer, with pCR achieved in 17 of 37 (46%) patients. The only study which failed to demonstrate a benefit of this group was conducted by Tfayli et al. [16] in 2020. In a phase I cohort of 15 patients with stage IB–IIIA predominantly adenocarcinoma NSCLC, the addition of avelumab to chemotherapy did not result in an improved overall response rate compared to historical controls. Whether this negative result is a reflection of pharmacodynamic differences in avelumab compared with other PD1/PD-L1 therapies, or due to the small cohort size is unknown.

## Neoadjuvant immunotherapy

Although most studies focus on chemoimmunotherapy as the preferred neoadjuvant approach to maximise response, there is data to demonstrate efficacy of immunotherapy as a monotherapy. The first trial data using neoadjuvant immunotherapy alone in resectable NSCLC emerged with initial results from Forde et al. [17] in 2018. A total of 20 patients completing 2 cycles of neoadjuvant nivolumab underwent radical surgery. There were no delays to surgery, no new safety signals, and a 45% MPR rate, with 90% achieving stable disease [17]. In light of these promising results, the same investigators opened a trial arm utilising neoadjuvant nivolumab with ipilimumab. Although this protocol demonstrated surgical feasibility, the study arm was discontinued after only 9 patients were enrolled due to toxicity, with 3 patients experiencing a grade ≥ 3 treatment related adverse events [18].

Arms 1 and 2 of NeoSTAR explored the efficacy of three doses of nivolumab, with arm 2 adding a single dose of ipilimumab in stage I–IIIA NSCLC [14]. 39 of 44 patients underwent curative surgery. The nivolumab and ipilimumab arm met its prespecified primary endpoint target of six MPRs, achieving this in 38% of patients (8/21). Furthermore, of those patients resected on trial, there was a higher pCR rate in the doublet arm (29% vs. 9%), without significant increase in toxicities. This highlights a promising role for combination checkpoint blockade in the neoadjuvant setting which may have an important place for those patients who are not suitable candidates for systemic chemotherapy. The benefit of combination therapy was also suggested in the NEOCOAST study which investigated durvalumab alone or in combination with other immune modulating therapies [19]. In patients who received neoadjuvant durvalumab alone, MPR rates were 11% but those who were treated with concomitant oleculumab (anti-CD73), monalizumab (anti-NKG2A) or danvatisern (anti-STAT3-antisense-oligonucleotide) had MPR rates of 19%, 30% and 31%, respectively. These collectively encouraging results support the need for further evaluation of combination immunotherapy in the neoadjuvant NSCLC setting.

More recently the IONESCO trial assessed the feasibility of three cycles of neoadjuvant durvalumab in patients with resectable NSCLC. Of the 43 patients proceeding to surgery, 8 (19%) achieved MPR, with 12-month OS and disease free survival (DFS) rates 89% and 78% respectively [20]. NEOMUN was a further phase II single-arm study which administered two cycles of pembrolizumab prior to surgery, with a comparable 27% MPR [21].

## Perioperative immunotherapy +/- chemotherapy

Perioperative immunotherapy, with and without chemotherapy, has been investigated in 7 recent trials. Of these, SAKK 14/16 was the earliest to be reported, assessing the utility of chemotherapy with cisplatin and docetaxel with sequential durvalumab using two cycles pre-operatively and 12 months in the adjuvant setting for stage IIIA NSCLC with N2 disease. This study yielded R0 resection rates of 93% (51/55, 93%) and a 73% 1-year EFS [22]. Following on from this, the efficacy of peri-operative durvalumab was reported by the AEGEAN investigators in a large phase III randomised trial comparing 12 cycles of post-operative durvalumab to placebo following 3 cycles of neoadjuvant chemo-immunotherapy in patients with stage II–IIIB resectable NSCLC [23]. The primary endpoints of this trials were EFS and PCR, in a modified intention to treat subset of 740 patients without actionable EGFR or ALK alterations. EFS was demonstrated in 73.4% of patients receiving durvalumab compared to 64.5% in the placebo group with a HR of 0.68, in addition to 17.2% vs. 4.3% pCR respectively [13% difference; 95% confidence interval (CI) 8.7 to 17.6; *P* < 0.001].

These results were very comparable to the largest of the perioperative trials, the phase III KEYNOTE-671 [24]. This randomized patients into perioperative pembrolizumab with chemotherapy vs. neoadjuvant chemotherapy alone. pCR rate was 18.1% in this study and 24-month EFS rates were 62.4% in the pembrolizumab group vs. 40.6% in the placebo arm (HR 0.58), although not yet enough to demonstrate a clear OS benefit at 2-years (80.9% vs. 77.6%). Interestingly, the highest pCR rates were achieved in the NADIM studies despite enrolling only stage IIIA patients. In the phase II NADIM trial, patients received 3 cycles of neoadjuvant carboplatin, paclitaxel and nivolumab, followed by surgery, then 12 months of adjuvant nivolumab. Of the 41 patients undergoing surgery, 34 (83%; 95% CI 68–93) had MPR, with 26 (63%) achieving pCR. The 3-year OS was 81.9% in the intention-to-treat population (Provencio et al. [25]). The NADIM II trial randomised patients to this same neoadjuvant chemoimmunotherapy regimen and compared it to chemotherapy alone. They also reported an excellent 37% pCR rate (although inferior to NADIM) in the combination group vs. 7% in the comparator arm (RR 5.34; 95% CI 1.34 to 21.23; *P* = 0.02) [25]. The only reason found to potentially account for the vast difference in pCR rates in the two NADIM trials was the higher carboplatin dose used in the latter (AUC6 vs. AUC5). The reasons for their superiority over CheckMate-816 [26] are interesting, though as yet unexplained.

## Summation

Overall, the results of the above trials support the clear role of neoadjuvant immunotherapy in early NSCLC. There is a distinct and demonstrable effect on enhancing pCR and MPR that is consistently observed in its use as monotherapy and in combination with chemotherapy, and extending into the perioperative setting. Furthermore, it offers higher rates of R0 resection without surgical delays, and does this with a tolerable side-effect profile. However, and perhaps of little surprise given the tempo of evolution in the field, there is significant heterogeneity across these trials in the selection of checkpoint inhibitor, the duration of their use pre- and post-operatively, and clinical endpoints measured.

## Safety of neoadjuvant immunotherapy

With additional agents being used in the cancer paradigm, especially in the curative setting, there is always concern about increasing toxicity. Recently, a meta-analysis of sixteen studies across multiple tumour streams demonstrated that grade ≥ 3 immune related adverse events was 24.0% with led to discontinuation of neoadjuvant protocols in 9.4% of patients [27]. In the NSCLC setting, a recent systematic review has shown no difference in grade 3–5 treatment related adverse events between patients receiving neoadjuvant chemoimmunotherapy vs. those receiving neoadjuvant chemotherapy only [28]. In fact, patients who had chemoimmunotherapy were more likely to have surgery and less likely to have progression of disease that prevented curative intent surgery. No differences in patient refusal for surgery has been demonstrated between chemoimmunotherapy and chemotherapy populations [28].

## Pathological responses as surrogate markers of survival

pCR is a key primary endpoint in a number of neoadjuvant studies and is defined as 0% residual viable tumour. Currently, the role of pCR as a confirmed surrogate endpoint of either recurrence free survival or OS is not well established. MPR defined arbitrarily as less than 10% residual viable tumour is an alternatively used measure [29]. Studies in the neoadjuvant chemotherapy and chemo-radiotherapy resectable NSCLC settings have supported the role of pCR as a predictive marker of OS [30, 31]. In 2020, a systematic review of neoadjuvant therapy of any kind in resectable lung cancer also supported the role of PCR as a surrogate marker of OS using 33 studies. They demonstrated that for OS by PCR status, the HRs ranged from 0.13 to 0.78, and the meta-analysed HR across 21 studies (6,672 patients) was 0.49 (95% CI 0.43–0.56) [32]. Recently, authors from the CheckMate-816 study investigated the influence of residual tumour volume (RVT) on EFS as defined by either recurrence or death by any cause [33]. They demonstrated that RVT was predictive of EFS with 2-year EFS rates of 90%, 60%, 57%, and 39% for patients with 0–5%, 5–30%, 30–80%, and > 80% RVT. This relationship was sustained despite the presence or absence of lymph node involvement of the original tumour. These results are the most recent in the neoadjuvant immunotherapy space and greatly support the use of PCR and RVT as appropriate surrogate markers of long-term progression free and OS.

## Predictive markers of immunotherapy response

**Figure 2:**
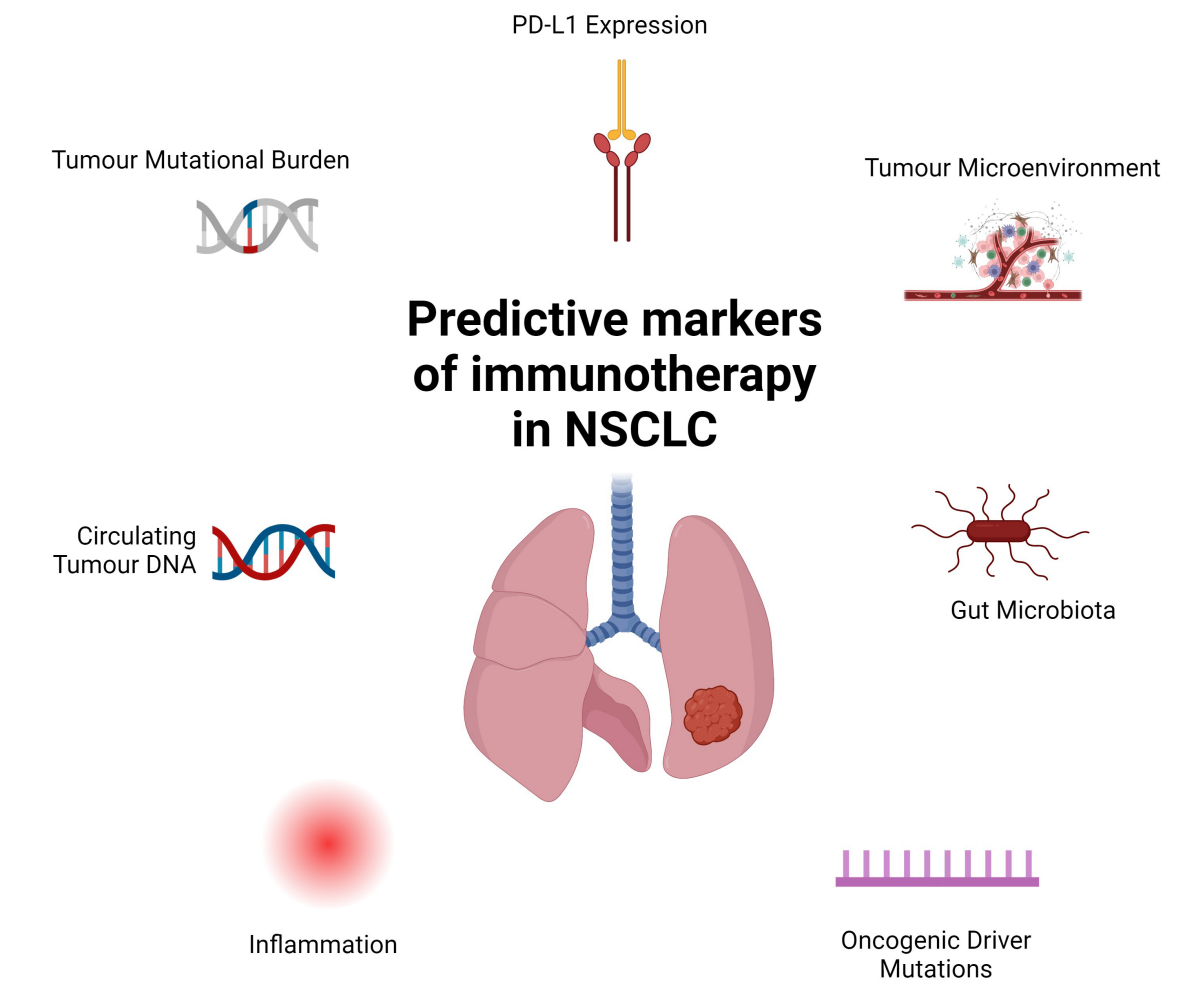
Predictive markers of immunotherapy in non-small cell lung cancer. Multiple predictive markers of immunotherapy have been investigated over the past few decades. Currently tumour mutational burden (TMB) and PD-L1 expression remain the two most clinically used markers but increasing data regarding the role of inflammation, gut microbiome and tumour microenvironment are emerging. Further, presence of oncogenic driver mutations such as EGFR and ALK also appear to dampen the likelihood of immunotherapy response in advanced NSCLC but their role as predictive markers in the neoadjuvant setting are currently unknown. ALK: anaplastic lymphoma kina. Created in BioRender. Nindra, U. (2024) BioRender.com/k45y858

The use of predictive molecular markers to identify patients with higher likelihood of response is of paramount importance and a number of these have been recognised in the literature and summarised in Figure 2. PD-L1 expression is recognised as an important indicator of immune-responsiveness in the metastatic setting [5]. Follow-up analysis of the LCMC3 cohort revealed a strong correlation between PD-L1 TPS and major pathological response [34]. The NEOMUN trial echoed these results, demonstrating an augmented pathologic response with pembrolizumab in patients with higher PD-L1 [21]. CheckMate-816 demonstrated that although benefit of nivolumab with chemotherapy was observed across all subgroups, the benefit was most pronounced in patients with PD-L1 expression of > 1%, although the trial was not statistically powered for this analysis [12]. A meta-analysis of neoadjuvant chemoimmunotherapy and chemotherapy in NSCLC similarly demonstrated benefit in EFS across all PD-L1 subgroups for chemoimmunotherapy [28]. However, a consistent theme observed across all of the above trials was that pCR is seen in patients with PD-L1 ≤ 1%. Although this might be explained by intratumoral heterogeneity, it highlights the need for further evaluation of the predictive ability of differential PD-L1 levels in prospective PD-L1 all-comer trials. In addition to PD-L1, tumour mutational burden (TMB) has also been shown to be a potential predictive biomarker for neoadjuvant immunotherapy in NSCLC [17]. Interestingly, in the same study, Forde et al. [17] did not note a correlation between TMB and PD-L1 expression thereby highlighting that these two biomarkers may act as independent predictive tools for clinicians in future. TMB has much more evidence in the metastatic NSCLC as being a predictive marker of immunotherapy response [35] and based on the KEYNOTE-158 study, the FDA has approved blanket use of pembrolizumab in patients with advanced cancer with high TMB [36].

The use of circulating tumour DNA (ctDNA) is becoming increasingly recognised as a promising surrogate marker for minimal residual disease and is gaining interest in the immunotherapy setting. The NADIM II trial demonstrated that low pre-treatment and post-treatment ctDNA levels were associated with significantly improved progression-free survival and OS [25]. An exploratory analysis of pre-operative ctDNA was also assessed in the CheckMate-816 cohort, which demonstrated a higher percentage of ctDNA clearance with nivolumab plus chemotherapy (56%; 95% CI 40–71) than with chemotherapy alone (35%; 95% CI 21–51), with a higher EFS in those patients achieving ctDNA clearance across both treatment arms [12]. Longitudinal assessment of ctDNA following resection of operable NSCLC has revealed that detectable ctDNA in the post-operative setting is associated with early recurrence and poor prognosis [37]. Moreover, the relapse risk in ctDNA negative patients did not change with receipt of adjuvant chemotherapy, heralding its potential utility as a tool to select patients suitable for adjuvant immunotherapy de-escalation in the future. This however requires further investigation of its use in dedicated perioperative immunotherapy trials.

There has also been increasing research into the role of gut microbiota and response to immune checkpoint inhibitors. Recently, it was shown that elevated diversity and composition of microbiota was correlated with improved response to immunotherapy [38]. Part of this response was thought to be due to presence of faecalibacterium that was accompanied by increased presence of short-chain fatty acids. Additionally, alterations in gut microbiota through the use of probiotics has also been correlated with improved immunotherapy response in NSCLC [39]. Whether this is a direct result of probiotic use or the result of changes in gut flora requires ongoing research. Given the suggestion of gut microbiota diversity, faecal transplantation has been postulated to augment immunotherapy response with some evidence in animal models of improved outcomes [38, 39]. However, further research in this space is required before human trials are explored.

## Conclusion

Overall, it is evident that based on multiple studies that concomitant neoadjuvant immunotherapy in addition to chemotherapy provides PCR and potential OS benefit. There is still an unmet need to identify patients who are most likely to benefit from therapy, as well as the ideal combination of immunotherapy regimen in the neoadjuvant setting. In addition to this, for those who do not achieve a PCR and therefore identified as high risk of recurrent disease, the optimal adjuvant treatment, including treatment intensification, remains unclear. Overall, the field of early lung cancer treatment is expanding and becoming increasingly complex and with neoadjuvant immunotherapy now becoming mainstay in other tumour streams such as breast cancer, increasing capacity to determine which patients would most benefit is essential. Future directions will need to be focused not only standardised treatment guidelines but also predictive clinical, biological, and potentially radiological markers of response and survival.

## Abbreviations

ALK: anaplastic lymphoma kina
CI: confidence interval
ctDNA: circulating tumour DNA
DFS: disease free survival
EFS: event free survival
EGFR: epidermal growth factor receptor
HR: hazard ratio
MPR: major pathological response
NSCLC: non-small cell lung cancer
OS: overall survival
pCR: pathological complete response
RVT: residual tumour volume
TMB: tumour mutational burden

## Declarations

### Availability of data and materials

Both Figures 1 and 2 were created using BioRender, please refer to the relevant section in the main text for specific information.

### Copyright

© The Author(s) 2024.
